# Risperidone-induced priapism in an autistic child: a case report

**DOI:** 10.1186/s13256-016-0956-x

**Published:** 2016-06-06

**Authors:** Bouchra Aabbassi, Abdeslam Benali, Fatima Asri

**Affiliations:** Research Team for Mental Health, University Hospital Mohamed VI, Marrakesh, Morocco

**Keywords:** Risperidone, Autistic child, Priapism, Side effect

## Abstract

**Background:**

Priapism is a prolonged stimulation with painful, persistent penile erection unaccompanied by sexual desire. It is a rare but serious urological emergency. Risperidone is an atypical antipsychotic widely prescribed for the treatment of behavior problems in children with autism spectrum disorder. It seems associated with priapism in children.

**Case presentation:**

We present a case of a 12-year-old Moroccan boy diagnosed with autism spectrum disorder who developed priapism while on an existing regimen of risperidone, and we report the treatment decisions that followed.

**Conclusions:**

Clinicians who prescribe risperidone should be aware of the possibility of this rare complication in their patients. Information about this possible side effect and instructions regarding appropriate response should be made available to caregivers of those in the at-risk group of young patients.

## Background

Priapism is a prolonged stimulation with painful, persistent penile erection unaccompanied by sexual desire. Priapism is a relatively rare condition; however, due to this potentially serious and long-term consequences and its potential as an adverse effect of many common medications, it is a matter of serious concern for clinicians. We present a case of an autistic child who developed priapism while on an existing regimen of risperidone, and we report the treatment decisions that followed.

## Case presentation

Our patient was a 12-year-old Moroccan boy diagnosed with autism spectrum disorder. In early childhood, he was unable to develop speech; he only screamed sharply if in distress or in need of attention. He avoided eye contact, and he often appeared to be looking into space, focusing on an unseen object. He had behavior problems, including hitting, kicking, and biting. He rarely played with other children, and he was unable to acquire new skills. He had no history of any major medical illness that could influence normal neurological development during childhood. Upon presentation to our clinic at 10 years of age, the boy could not utter a word, but only shouted and screamed. He had not been exposed to any form of schooling and appeared distant when attempts were made to interact with him. The child met the criteria of the *Diagnostic and Statistical Manual of Mental Disorders, Fourth Edition,* for a diagnosis of autism spectrum disorder.

At the age of 12 years, the boy’s associated behavior problems included destructive tendencies, screaming without apparent reason, aggression, self-injury, and irritability. Risperidone was prescribed to manage these disruptive behavior symptoms. The dosage was increased gradually to 2 mg/day to control the boy’s behavior problems. After taking the medication, the child awoke with a painful erection that lasted 6 h. He had no history of penile, genital, or pelvic trauma, and he had no evidence of any infection or malignancy. He had no change in his current medications and no reported use of any medication or any herbal preparation.

He was immediately sent to the emergency department. Laboratory tests were performed, including a complete blood count, a basic metabolic profile, and a coagulation study. All the results were within normal limits. Discontinuation of risperidone led to an improvement in the boy’s pain. The urology service was consulted, and the boy’s priapism resolved completely within a few hours after intracavernosal washing. Risperidone was the only known causative factor, and it was discontinued. On leaving the hospital, a prescription of antipsychotic treatment was indicated. We prescribed aripiprazole; however, the boy’s family did not have the financial means to procure this expensive medication. Instead, the boy was treated with 150 mg/day of sulpiride safely with a favorable outcome. The boy’s priapism had not returned when he was seen in follow-up 4 months later. In this patient, we established a probable causal relationship between risperidone and priapism using the Naranjo Adverse Drug Reaction Probability Scale, on which the patient’s score was 7 [1].

## Discussion

Priapism can occur in all age groups. The pathophysiology is still unclear, and it is considered to be multifactorial in origin [2]. Some common etiological factors in children include leukemia, spinal and perineal trauma, sickle cell anemia, thrombocytopenia, malignancy, neurological disorders, and the use of certain drugs [2, 3]. Antipsychotic agents are implicated in 15–26 % of cases of priapism associated with medications [3]. Among atypical antipsychotics, clozapine, risperidone, and olanzapine have been reported to be associated with the condition as well [2–4]. Recent evidence indicates that atypical antipsychotics represent a promising option for the treatment of autism spectrum disorder [2, 3]. In particular, risperidone appears to be effective in treating aggressiveness, hyperactivity, irritability, stereotypies, social withdrawal, and lack of interests [5]. However, risperidone can have side effects that suggest an informal analysis of the medication’s use. Much has been discussed about the metabolic side effects; however, relatively little is known about the occurrence of ischemic priapism, which has been reported with risperidone in a few cases in different countries [3, 4, 6, 7].

Ischemic priapism accounts for 95 % of cases of priapism [2]. It is caused by obstruction of the venous drainage from the corpora cavernosa of the penis. Although the relationship between psychotropic medication and priapism is well documented, the exact incidence is unknown. The proposed mechanism responsible for this obstruction is increased parasympathetic tone relative to sympathetic tone due to direct α-adrenergic receptor blockade [2, 4]. Risperidone is a potent α_1_- and α_2_-adrenergic receptor antagonist and therefore could potentially cause priapism [2].

Priapism may occur at any time during the treatment course of psychotropic medications, even without a change of dosage and even as monotherapy [2, 6]. It is considered a serious urological emergency. It leads to vessel stasis, hypoxia, ischemia, and acidosis, resulting in irreversible cavernosal fibrosis [2, 7]. Impotence has been reported in more than 50 % of the cases in which priapism was not treated [2, 7]. Treatments including ice packs, enemas, medications, and anesthesia generally do not produce consistent results. Management usually includes intracavernosal washing followed by intracavernosal injection of an α-adrenergic agonist. If no α-adrenergic agonist is effective, shunt surgery is performed between the corpora cavernosa and the corpus spongiosum; otherwise, saphenocavernosal surgery is suggested [2, 5, 7]. If the patient needs to be maintained on an antipsychotic regimen, the dosage should be decreased or the medication should be discontinued and replaced together with a medical follow-up, given the high degree of risk for priapism with antipsychotics [2, 3, 6]. Risperidone and aripiprazole are currently the only drugs approved by the U.S. Food and Drug Administration for the treatment of behavior problems in children with autism spectrum disorder [6]. If appropriate, it is generally recommended to switch to another antipsychotic with fewer α_1_-blocking properties, such as sulpiride [8].

## Conclusions

Clinicians who prescribe risperidone should be aware of the possibility of the rare complication of priapism in their patients. The case of our patient is of particular interest, given both the need for prompt recognition of this emergency and the common nonverbal and/or noncommunicative features of autism. Information about this possible side effect and instructions regarding appropriate response should be made available to caregivers of those in the risk group of young patients.
